# Identify Molecular Mechanisms of Jiangzhi Decoction on Nonalcoholic Fatty Liver Disease by Network Pharmacology Analysis and Experimental Validation

**DOI:** 10.1155/2020/8829346

**Published:** 2020-12-12

**Authors:** Lei Wang, Yin Zhi, Ying Ye, Miao Zhang, Xing Ma, Hongyun Tie, Xiaokun Ma, Ni Zheng, Wei Xia, Yanan Song

**Affiliations:** The Seventh People's Hospital of Shanghai University of Traditional Chinese Medicine, 358 Datong Road, Pudong, Shanghai 200137, China

## Abstract

**Background:**

Jiangzhi Decoction (JZD), a traditional herb mixture, has shown significant clinical efficacy against nonalcoholic fatty liver disease (NAFLD). However, its multicomponent and multitarget characteristics bring difficulty in deciphering its pharmacological mechanisms. Our study is aimed at identifying the core molecular mechanisms of JZD against NAFLD.

**Methods:**

The active ingredients were searched from Traditional Chinese Medicine Systems Pharmacology (TCMSP) database and Traditional Chinese Medicine Integrated Database (TCMID). The targets of those ingredients were identified using ChemMapper database based on 3D structure similarity. NAFLD-related genes were searched from DisGeNET database and Gene Expression Omnibus (GEO) database. Then, we performed protein-protein interaction (PPI) analysis, functional enrichment analysis, and constructed pathway networks of “herbs-active ingredients-candidate targets” and identified the core molecular mechanisms and key active ingredients in the network. Also, molecular docking was carried out to predict the ligands of candidate targets using SwissDock. Finally, the human hepatic L02 cell line was used to establish the NAFLD model *in vitro.* The effect and key molecules were validated by Oil Red O staining, biochemical assays, and quantitative real-time PCR (qRT-PCR).

**Results:**

We found 147 active ingredients in JZD, 1285 targets of active ingredients, 401 NAFLD-related genes, and 59 overlapped candidate targets of JZD against NAFLD. 22 core targets were obtained by PPI analysis. Finally, nuclear receptor transcription and lipid metabolism regulation were found as the core molecular mechanisms of JZD against NAFLD by functional enrichment analysis. The candidate targets PPAR*α* and LXR*α* were both docked with hyperin as the most favorable interaction, and HNF4*α* was docked with linolenic acid ethyl ester. According to *in vitro* experiments, it was found that JZD had an inhibitory effect on lipid accumulation and regulatory effects on cholesterol and triglycerides. Compared with OA group, the mRNA expression levels of *PPARα* and *HNF4α* were significantly upregulated in JZD group (*P* < 0.05), and *LXRα* was significantly downregulated (*P* < 0.001).

**Conclusion:**

JZD might alleviate hepatocyte steatosis by regulating some key molecules related to nuclear receptor transcription and lipid metabolism, such as *PPARα*, *LXRα*, and *HNF4α*. Our study will provide the scientific evidences of the clinical efficacy of JZD against NAFLD.

## 1. Introduction

Nonalcoholic fatty liver disease (NAFLD) encompasses a spectrum of liver pathology that is characterized by the excessive accumulation of fat in the liver, including simple steatosis nonalcoholic fatty liver, nonalcoholic steatohepatitis (NASH), steatofibrosis, cirrhosis, and hepatocellular carcinoma [1]. In the face of a global obesity epidemic, NAFLD has emerged as the most common form of chronic liver disease, affecting an estimated 25% of the general population worldwide [2, 3]. Epidemiological researches have reported that NAFLD is one of the three main causes of cirrhosis [4], and NASH is rapidly becoming the leading cause of end-stage liver disease [5, 6]. However, except for lifestyle interventions, such as exercise and dietary, there is still no approved pharmacotherapy for NAFLD. Therefore, it is important to explore and develop valid pharmacotherapies for NAFLD [7].

In recent years, traditional Chinese medicine (TCM) has been proved effective in the case of alone or integrated with western medicine and has attracted more and more people's attention [8, 9]. Jiangzhi Decoction (JZD), a clinically used herbal formula developed in accordance with TCM pathogenesis, is composed of the following five medicinal herbs: *Trichosanthes kirilowii* Maxim. (TK), *Alisma orientale* (Sam.) Juzep (AO), *Angelica sinensis* (Oliv.) Diels (AS), *Crataegus pinnatifida* Bge. (CP), and *Polygonum multiflorum* Thunb. (PM) (Table 1). Previous evidence has proved the efficacy of JZD on regulating lipid metabolism [10]. However, the molecular mechanisms of JZD are still unclear and need further exploration.

**Figure 1:**
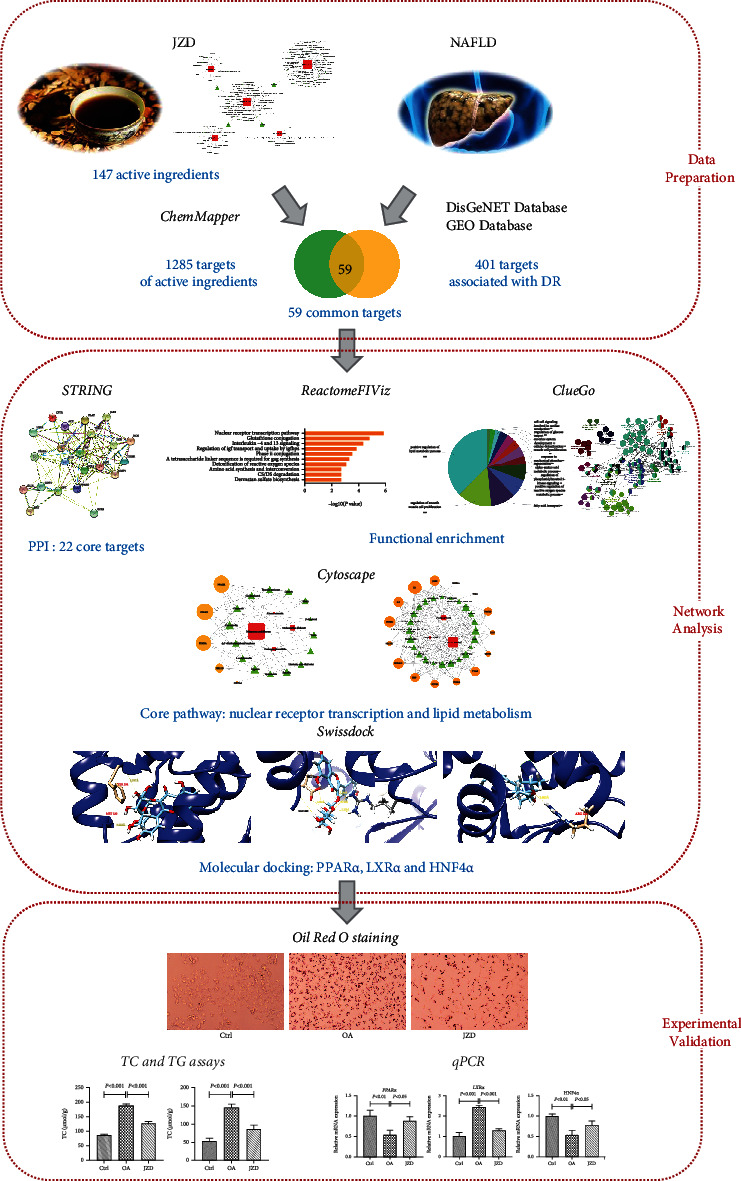
The flowchart for exploring the molecular mechanisms of JZD against NAFLD.

Because Chinese herbal medicines have the characteristics of multicomponent and multitargeted effects, conventional strategies can be hardly used to explore their pharmacological mechanisms. Network pharmacology, a novel approach based on systems biology, has been proved suitable for analyzing the complex relationships of various ingredients and effects in Chinese herbal medicines [11]. In this study, network pharmacology was carried out to investigate the molecular mechanisms according to screening a great deal of candidate ingredients, predicting multiple drug targets, analyzing possible signaling pathways, conducting herbs-ingredients-targets networks, and predicating the possible ligands for candidate targets. Also, the NAFLD model *in vitro* was established to validate the inhibitory effect on lipid accumulation and the changes of expression levels of key molecules in JZD against NAFLD (Figure 1).

## 2. Methods

### 2.1. Data Preparation

#### 2.1.1. Searching for Active Ingredients of JZD

The active ingredients of JZD were collected from two databases. One is Traditional Chinese Medicine Systems Pharmacology (TCMSP) database [12] (http://lsp.nwu.edu.cn/tcmsp.php), which contains large number of herbal entries, drug-disease networks, and drug-target networks. A great deal of herbal information can be obtained from TCMSP database, including ingredients, molecule name, molecular weight (MW), drug-likeness (DL), human oral bioavailability (OB), half-life (HL), water partition coefficient (AlogP), number of hydrogen bond donors and receptors (Hdon/Hacc), Caco-2 permeability (Caco-2), and blood-brain barrier (BBB). The active ingredients of JZD were screened out according to the ADME parameter, and the ingredients with DL ≥ 0.18 were regarded as active ingredients [13].

If the herbal information could not be found in TCMSP database, the other database would be used. It is Traditional Chinese Medicine Integrated Database (TCMID) (http://www.megabionet.org/tcmid/), which contains 46929 prescriptions, 8159 herbs, 43413 total ingredients, 8182 drugs, 4633 diseases, 1045 prescription ingredients, 778 herbal mass spectra, and 3895 mass spectrometry of ingredients [14]. By combining the information from two databases above, the active ingredients of JZD were identified.

#### 2.1.2. Identification of Targets of Active Ingredients

ChemMapper database (http://lilab.ecust.edu.cn/chemmapper/) is a versatile web server for exploring pharmacology and chemical structure association based on molecular 3D similarity method [15]. We searched the predicted targets of each active ingredient in JZD from ChemMapper database and screened according to the criteria of 3D structure similarity above 1.0 and prediction score above 0 [16]. The full names of targets were converted to gene symbol based on the UniProt ID in UniProt database (http://www.uniprot.org/) for further analysis.

#### 2.1.3. Searching for NAFLD-Related Genes

DisGeNET database (http://www.disgenet.org/web/DisGeNET/menu/home) is a knowledge management platform integrating and standardizing data about disease-associated genes and variants from multiple sources, including the scientific literature [17]. Known genes of NAFLD were searched from DisGeNET database using “nonalcoholic fatty liver disease” as the keyword, and the top 30% of genes were regarded as important genes for further analysis.

In addition, Gene Expression Omnibus (GEO) database (http://www.ncbi.nlm.nih.gov/geo/) is an international public repository for high-throughput microarray and next-generation sequence functional genomic data sets submitted by the research community [18]. The main differentially expressed genes (DEGs) between mild NAFLD and advanced NAFLD were extracted from microarray data GSE31803 and GSE49541 in GEO database, with a cutoff value of *P* < 0.05 and fold change ∣FC | ≥3.

### 2.2. Network Analysis

#### 2.2.1. Protein-Protein Interaction (PPI) Analysis

The overlapped genes between the target genes of active ingredients and NAFLD-related genes were imported into the STRING database to construct PPI network. STRING database integrates the quality-controlled protein-protein association networks of a large number of organisms. We selected the core PPI targets according to the degree score above the average value and the confidence score above 0.9.

#### 2.2.2. Functional Enrichment Analysis

ReactomeFIViz and ClueGO, two kinds of plug-ins for Cytoscape, were used to perform functional enrichment analysis. ReactomeFIViz is a highly reliable protein functional interaction network covering around 60% of total human genes based on Reactome database, the most popular and comprehensive open-source biological pathway knowledgebase [19]. ClueGO integrates Gene Ontology (GO) terms and KEGG/BioCarta pathways and creates a functionally organized GO/pathway term network [20]. *P* < 0.01 was regarded as the significant cutoff in this study.

#### 2.2.3. Pathway Network Construction

Pathway networks of “herbs-active ingredients-candidate targets” were constructed using the Cytoscape 3.3.0 software. The Network Analyzer plug-in was used to identify key active ingredients and critical candidate targets based on the criterion below: nodes with degree values exceeding the average value of all nodes in the network. The degree value is the number of edges a node has in a network, which indicates how many herbs/ingredients/targets one node is related with. If the degree value of a node is larger, the node is believed to play a more important role in the network.

#### 2.2.4. Molecular Docking

Molecular docking was carried out online using SwissDock (http://swissdock.ch/) web service. Crystal structures of candidate targets in PDB format and relative ligands in MOL2 format were uploaded. The lowest Gibbs free energy (△*G*) was predicted in silico. The UCSF Chimera 1.14 software was used for visualizing the results and creating 3D images.

### 2.3. Experimental Validation

#### 2.3.1. Cell Culture and Treatment

Human hepatic L02 cell lines were cultured in RMPI medium containing 10% fetal bovine serum (FBS) (Gibco, Thermo Fisher Scientific, Inc., Waltham, MA, USA) and incubated at 37°C in 5% CO2. Cells without any treatments were used as control. The cells were treated with 0.2 mM oleic acid (OA) for 24 h to establish the NAFLD model *in vitro*. Afterward, JZD (500 *μ*g/ml) was added into the medium. After another 24 h of incubation, the cells were analyzed.

#### 2.3.2. Oil Red O Staining

Briefly, the cells were fixed with 4% paraformaldehyde. After three times washing with PBS, the culture plates of the cells were added into 60% isopropanol and allowed to stand for 5 min. Freshly diluted oil red working solution was applied to cells for 15 min. After rinsing with 60% isopropanol, the cells were counterstaining with hematoxylin. Finally, the pictures were captured with a light microscope (OLYMPUS, Japan) at ×200 magnification.

#### 2.3.3. Cholesterol (TC) and Triglyceride (TG) Assays

Cells from the different groups were harvested and washed twice with PBS. The intracellular TC and TG were measured using the biochemistry assay kits in accordance with the manufacturer's instruction (Jiancheng, Nanjing, China). The TC and TG concentrations were normalized to the total cell protein concentration.

#### 2.3.4. Quantitative Real-Time PCR (qRT-PCR)

Total RNA was extracted using TRIzol Reagent (Invitrogen, Carlsbad, CA, USA). The quality of RNA was measured by Nanodrop 2000 (Thermo Scientific, Rockford, IL, USA), and equal amounts of RNA were reverse-transcribed into cDNA using First-Strand cDNA Synthesis kits (Invitrogen, Carlsbad, CA, USA). qRT-PCR was carried out using ABI 7500 System (Applied Biosystems, Foster City, CA, USA) under the following parameters: 95°C for 30 s, 95°C for 5 s (40 cycles), 60°C for 30 s, and 72°C for 15 s. The gene primer pairs used in this study were as follows: 5′-TCCGACTCCGTCTTCTTGAT-3′ and 5′-GCCTAAGGAAACCGTTCTGTG-3′ for *PPARα*, 5′-TGGACACCTACATGCGTCGCAA-3′ and 5′-CAAGGATGTGGCATGAGCCTGT-3′ for *LXRα*, 5′-CAGGCTCAAGAAATGCTTCC-3′ and 5′-GGCTGCTGTCCTCATAGCTT-3′ for *HNF4α*, and 5′-CGGAGTCAACGGATTTGGTCGTAT-3′ and 5′-AGCCTTCTCCATGGTGGTGAAGAC-3′ for *GAPDH*. The threshold cycle (Ct) of each gene was normalized to *GAPDH* mRNA, and the fold change was calculated by 2^-△△Ct^ method. Each sample was run three times at least.

### 2.4. Statistical Analysis

The quantitative data were presented as mean ± standard deviation (SD). One-way ANOVA analysis, followed by Dunnett post hoc test, was used to determine significant differences between different groups using the SPSS software (version 21.0, Chicago, IL, USA). *P* < 0.05 was considered statistically significant.

## 3. Results

### 3.1. Candidate Targets of JZD against NAFLD

#### 3.1.1. Active Ingredients of JZD

**Figure 2:**
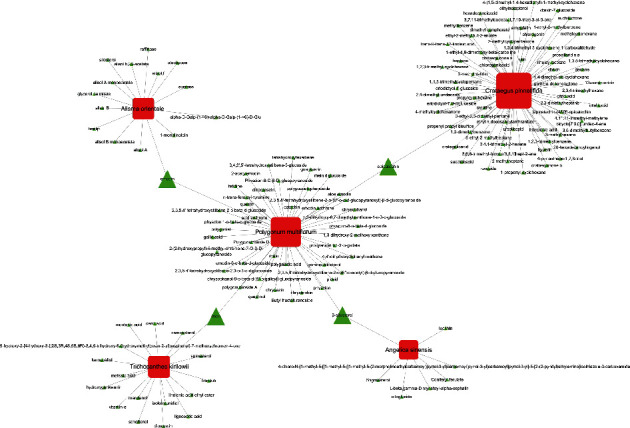
147 active ingredients found in JZD. The red square represents herbs in JZD, and green triangle represents ingredients of those herbs.

Among the five main herbs of JZD, TK, AO, and AS were searched from TCMSP database, while the other herbs, CP and PM, could not be found in TCMSP database and were searched from TCMID database. Based on the criteria of DL ≥ 0.18, a total of 147 active ingredients were finally screened out in this study (Figure 2 and Supplementary Table 1). The numbers of active ingredients in TK, AO, AS, CP, and PM were 17, 15, 7, 66, and 46, respectively. There were four ingredients overlapped in two herbs, including emodin in AO and PM, epicatechin in CP and PM, tricin in TK and PM, and *β*-sitosterol in AS and PM.

A note about those results was that ADME parameters were not provided in TCMID database. Thus, the ingredients in CP and PM did not be filtered according to the ADME parameters. Maybe it was the reason why the amounts of ingredients in CP and PM were much larger than those in TK, AO, and AS.

#### 3.1.2. Targets of Active Ingredients of JZD

The direct targets of each chemical ingredient in JZD were identified from ChemMapper database. According to the criteria of 3D structure similarity above 1.0 and prediction score above 0, a total of 1285 targets of active ingredients of JZD were obtained (Supplementary Table 2). Our analysis showed that gallic acid, citric acid, and succinic acid were the top three active ingredients targeting 525, 481, and 440 targets, respectively.

#### 3.1.3. Genes Related to NAFLD

A total of 333 known genes were found from DisGeNET database, and the top 30%, that were 100 genes, were chosen for further analysis. Based on the cutoff value of *P* < 0.05 and fold change ∣FC | ≥3, a total of 315 DEGs were extracted from microarray data GSE31803 and GSE49541. After duplicates of genes from DisGeNET database and GEO data were eliminated, a total of 401 genes were identified as NAFLD-related genes (Supplementary Table 3).

#### 3.1.4. Targets of JZD against NAFLD

According to the search and analysis above, we obtained a total of 59 overlapped genes between the target genes of active ingredients and NAFLD-related genes, which were predicted as the candidate targets of JZD against NAFLD (Table 2).

### 3.2. Key Targets and Signaling Pathway of JZD against NAFLD

#### 3.2.1. Core Targets in Protein-Protein Interaction (PPI)

**Figure 3:**
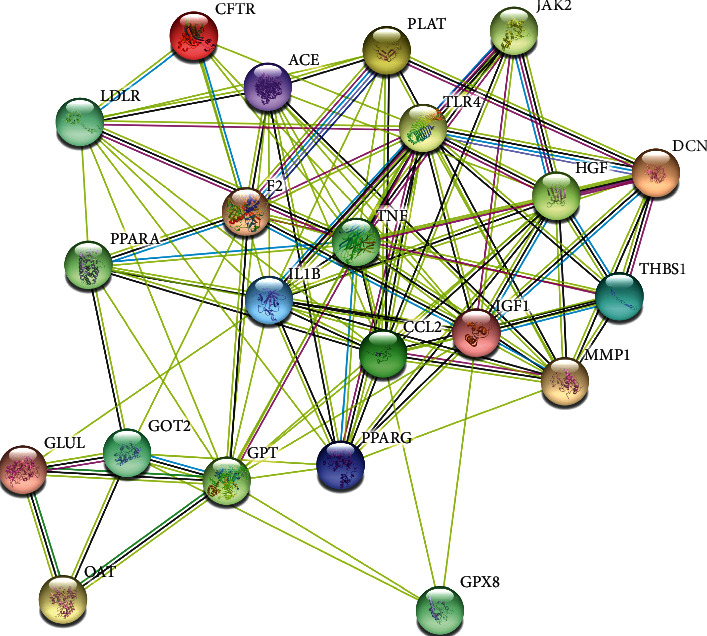
PPI network of 22 core targets of JZD against NAFLD. In the PPI diagram, each solid circle represents a target, and the middle of the circle shows the structure of the protein.

The 59 candidate targets were imported into the STRING database to construct the PPI network. According to the screening criteria of the degree score above the average value and the confidence score above 0.9, 22 core targets were obtained, including TNF, IGF1, IL1B, GPT, CCL2, HGF, TLR4, PPARG, GOT2, LDLR, MMP1, F2, DCN, ACE, GLUL, PPARA, THBS1, JAK2, CFTR, PLAT, OAT, and GPX8 (Figure 3). The 22 core targets may be the potential targets in the treatment of JZD against NAFLD.

#### 3.2.2. Functional Enrichment Analysis of the Overlapped Genes

**Figure 4:**
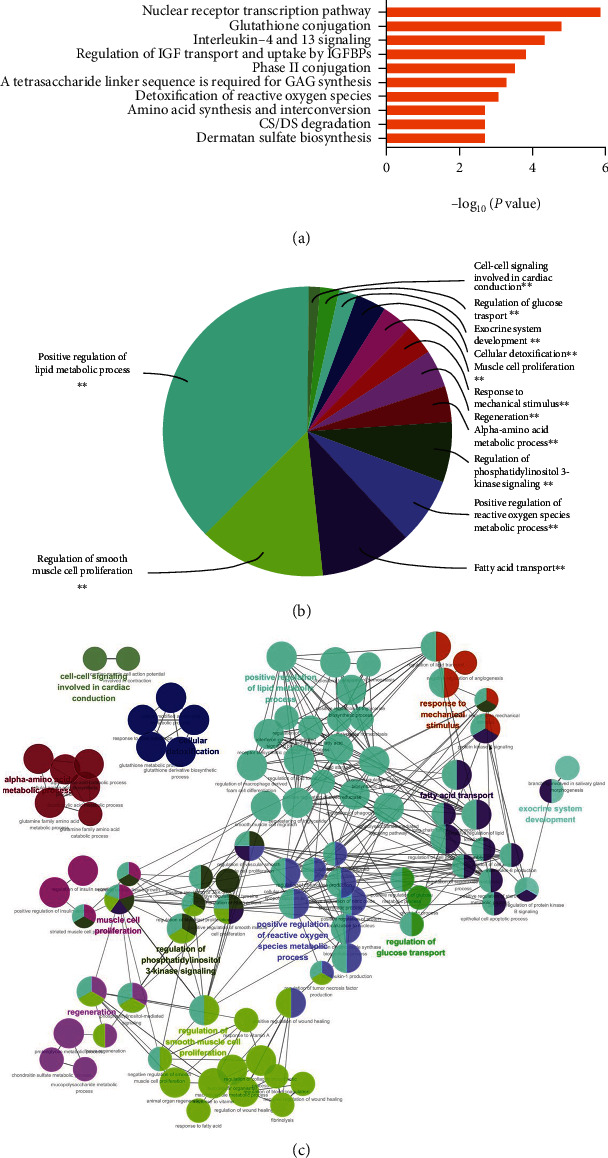
Functional enrichment analysis from ReactomeFIViz and ClueGO. (a) The column bar graph from ReactomeFIViz. It shows the top 10 significantly related pathways, including nuclear receptor transcription pathway, glutathione conjugation, interleukin-4 and 13 signaling, regulation of insulin-like growth factor (IGF) transport and uptake by insulin-like growth factor binding proteins (IGFBPs), and phase II conjugation. (b) The pie chart from ClueGO. It shows the enriched signaling pathway categories based on the kappa coefficient, including positive regulation of lipid metabolic process, regulation of smooth muscle cell proliferation, fatty acid transport, positive regulation of reactive oxygen species metabolic process, regulation of phosphatidylinositol 3-kinase signaling, and alpha-amino acid metabolic process. (c) The functional enrichment network from ClueGO. The node represents the signaling pathway, and the size of each node represents the enrichment significance of each signaling pathway. The larger the node is, the more significant the pathway is. The line represents the correlation between functions, and the thickness of each line represents the kappa coefficient between functions. The thicker the line is, the greater the kappa coefficient is.

ReactomeFIViz and ClueGO pathway analyses were further performed for the overlapped genes. The top 10 significantly related pathways were shown in Figure 4(a) by ReactomeFIViz analysis, including nuclear receptor transcription pathway, glutathione conjugation, interleukin-4 and 13 signaling, regulation of insulin-like growth factor (IGF) transport and uptake by insulin-like growth factor binding proteins (IGFBPs), and phase II conjugation. At the same time, according to ClueGo analysis, signaling pathways were divided into 13 enriched categories based on the kappa coefficient, including positive regulation of lipid metabolic process, regulation of smooth muscle cell proliferation, fatty acid transport, positive regulation of reactive oxygen species metabolic process, regulation of phosphatidylinositol 3-kinase signaling, alpha-amino acid metabolic process, regeneration, response to mechanical stimulus, muscle cell proliferation, cellular detoxification, exocrine system development, regulation of glucose transport, and cell-cell signaling involved in cardiac conduction (Figures 4(b) and 4(c)). The functional enrichment analysis indicated that nuclear receptor transcription and lipid metabolism regulation might play an important role in the treatment of JZD against NAFLD.

#### 3.2.3. Pathway Network Construction of Herb-Ingredient-Target

**Figure 5:**
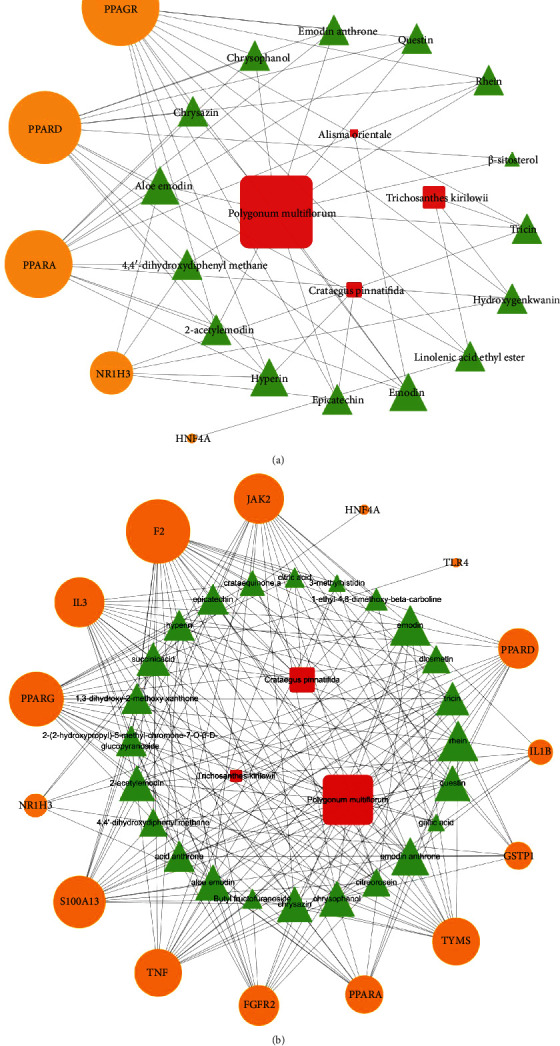
The top significantly related pathways of “herb-ingredient-target” networks in JZD against NAFLD: (a) nuclear receptor transcription pathway; (b) regulation of lipid metabolic process. The red square represents herbs in JZD, green triangle represents ingredients of those herbs, and orange circle represents candidate targets of JZD against NAFLD.

To identify key molecular mechanisms of JZD against NAFLD, we further constructed herb-ingredient-target networks based on the top significantly related pathways from functional enrichment analysis (Figure 5). We found that 12 ingredients appeared in both nuclear receptor transcription pathway and lipid metabolism regulation pathway, including hyperin, emodin, emodin anthrone, questin, rhein, tricin, aloe emodin, 2-acetylemodin, epicatechin, chrysazin, chrysophanol, 4,4′-dihydroxydiphenyl methane. The results implied that those key active ingredients all participated in nuclear receptor transcription and lipid metabolism regulation, which might be the key molecular mechanisms of JZD against NAFLD.

#### 3.2.4. Molecular Docking of Candidate Targets

**Figure 6:**
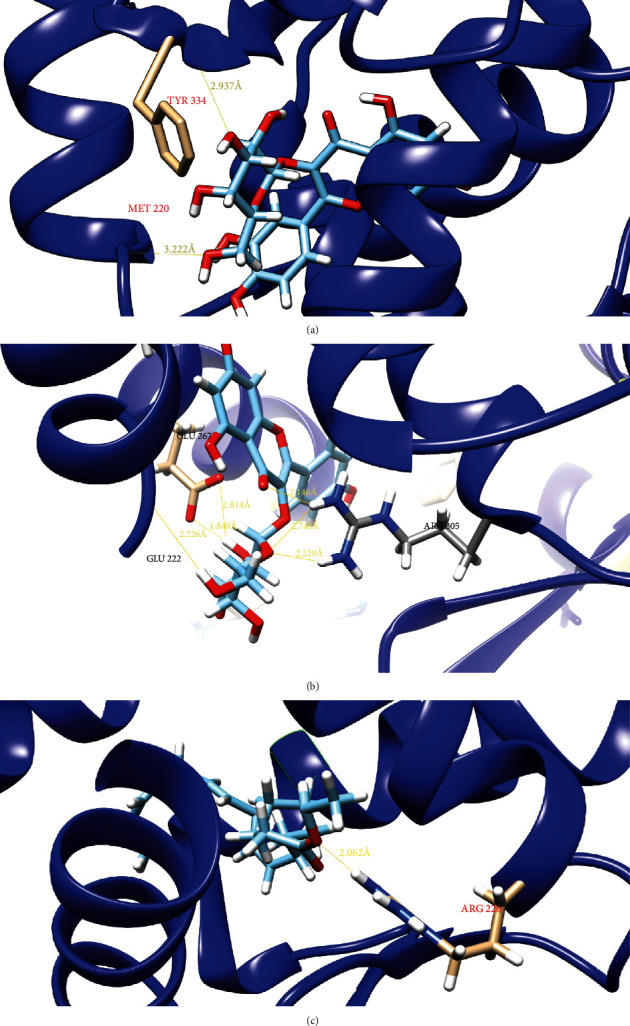
The PPAR*α*, LXR*α*, and HNF4*α* structure models docked with corresponding ligands. The protein structures are visualized with the ribbon model. The residues and the ligands are visualized with the stick model. (a) PPAR*α* was docked with hyperin at the binding sites of TYR 334 and MET 220 residues; (b) LXR*α* was also docked with hyperin at the binding sites of GLN 222, GLU 267, and ARG 305 residues; (c) HNF4*α* was docked with linolenic acid ethyl ester at the binding sites of ARG 226 residues.

According to the pathway network construction of herb-ingredient-target, we chose the candidate targets both in above two networks and closely related with liver lipid regulation that were PPAR*α*, LXR*α*, and HNF4*α*. Based on Swissdock calculation, hyperin showed estimated △*G* of -10.13 kcal/mol and -8.91 kcal/mal for PPAR*α* and LXR*α*, respectively, as the most favorable interaction, and linolenic acid ethyl ester showed △*G* of -8.49 kcal/mol for HNF4*α*.  Figure 6 shows the visualization of the most energetically favorable binding of the ligands into the protein PPAR*α*, LXR*α*, and HNF4*α*. As shown in Figure 6, PPAR*α* was docked with hyperin at the binding sites of TYR 334 and MET 220 residues, LXR*α* was also docked with hyperin at the binding sites of GLN 222, GLU 267, and ARG 305 residues, and HNF4*α* was docked with linolenic acid ethyl ester at the binding sites of ARG 226 residues.

### 3.3. Experimental Validation of JZD against NAFLD *in vitro*

#### 3.3.1. The Inhibitory Effect of JZD on Lipid Accumulation

**Figure 7:**
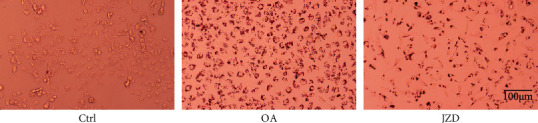
The inhibitory effect of JZD on lipid accumulation. Representative of Oil Red O staining in L02 cells incubated in the control group, OA group (2 mM OA), and JZD group (2 mM OA and 500 *μ*g/ml JZD). Magnification: ×200.

After L02 cells were treated with 0.2 mM OA for 24 h, epithelial morphology of hepatocytes was found to be transformed into bulky lipid laden round cells, stained red due to Oil Red O stain. Compared with the OA group without JZD treatment, there was a significant reduction in the lipid content of steatotic hepatocytes in the JZD group, indicating that JZD had an inhibitory effect on lipid accumulation (Figure 7).

#### 3.3.2. The Effects of JZD on TC and TG

**Figure 8:**
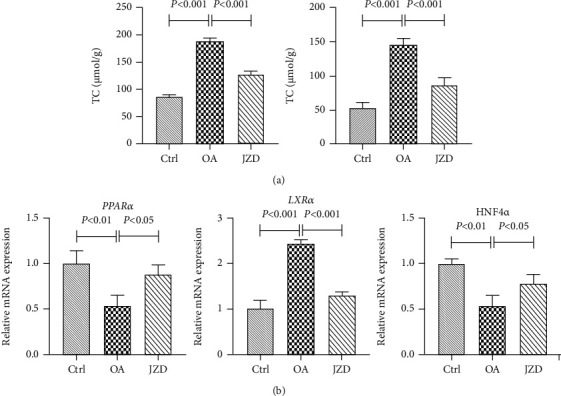
TC and TG contents (a) and relative mRNA expression levels of key genes (b) in JZD against NAFLD. *N* = 3 in each group. Values were expressed as mean ± standard deviation (SD). Significant differences were analyzed by one-way ANOVA with Dunnett post hoc test.

Compared with the control group, the contents of TC and TG statistically increased in the OA group (*P* < 0.001). Compared with the OA group, the contents of TC and TG significantly decreased in the JZD group (*P* < 0.001) (Figure 8(a)). It showed the regulatory effects of JZD on TC and TG.

#### 3.3.3. Changes of Expression Levels of Key Molecules in JZD against NAFLD

Compared with the control group, the mRNA expression levels of *PPARα* and *HNF4α* were significantly downregulated in the OA group (*P* < 0.01), and *LXRα* was significantly upregulated (*P* < 0.001). Compared with the OA group, the mRNA expression levels of *PPARα* and *HNF4α* were significantly upregulated in the JZD group (*P* < 0.05), and *LXRα* was significantly downregulated (*P* < 0.001) (Figure 8(b)). It implied us that JZD might alleviate hepatocyte steatosis by regulating the mRNA expression of these key molecules from network pharmacology analysis.

## 4. Discussion

NAFLD is characterized by abnormal lipid metabolism and excessive lipid accumulation in hepatocytes. Most of the herbs in JZD have been reported to take part in the regulation of lipid metabolism in NAFLD or other related diseases. Some researchers showed that AO could prevent hepatic triglyceride accumulation through suppressing de novo lipogenesis and increasing lipid export and control oxidative stress markers, lipoapoptosis, liver injury panels, and inflammatory and fibrotic mediators, eventually influencing steatohepatitis and liver fibrosis [21, 22]. The components of AS have been proved to regulate lipid and glucose metabolism [23–25]. Liu et al. found that a diet formula of CP and three other herbs could alleviate hepatic steatosis and insulin resistance *in vivo* and *in vitro* [26]. Yu et al. performed a series of experiments to confirm that the active components of PM could promote the lipolysis of cholesterol and triglyceride, increase the content of HTGL, and reduce LDL and VLDL [27–29]. However, the synergetic mechanisms of all the herbs in JZD were still unclear.

In our study, nuclear receptor transcription and lipid metabolism regulation were found as the core pathways which JZD mainly participated in when alleviating NAFLD. As we know, there are 48 nuclear receptors categorized into 7 subfamilies designated as NR0-NR6 [30]. Of particular importance in NAFLD are specific members of NR1 subfamily [31]. Most potential targets of JZD in Figure 2 belong to NR1 subfamily, including PPARs (peroxisome proliferator-activated receptors, PPARA/PPAR*α*, PPARD/PPAR*β*/*δ*, PPARG/PPAR*γ*; NR1C1-3) and LXR*α* (liver X receptor *α*; NR1H3). PPAR*α* activation induces the increase in fatty acid oxidation, ketogenesis, and gluconeogenesis [32]. PPAR*β*/*δ* activation exerts regulatory effects on fatty acid catabolism, reverses cholesterol transport and energy metabolism, and even reduces insulin resistance and plasma glucose [33]. PPAR*γ* shifts lipids from nonadipose organs such as the liver and skeletal muscles to white adipose tissue, leading to the attenuation of lipotoxicity [34]. In general, PPAR activation is thought to be beneficial in NAFLD, and clinical trials of single/dual receptor agonists are underway [31]. Another important nuclear receptor LXR*α* acts as the negative regulator of cholesterol metabolism through the induction of hepatocyte cholesterol catabolism, excretion, and the reverse cholesterol transport pathway [35]. Furthermore, HNF4A/HNF4*α* (hepatocyte nuclear factor 4*α*; NR2A1) also belongs to the subfamily of nuclear receptors. Previous study reported that HNF4*α* could prevent liver steatosis by controlling hepatic carboxylesterase 2 expression and modulating lipolysis, lipogenesis, and endoplasmic reticulum in NAFLD [36]. Therefore, they are all potential therapeutic targets for the treatment of NAFLD.

Our research found that some ingredients in JZD might be the key ones for NAFLD treatment, such as emodin, hyperin, and rhein. Many previous studies have reported that those ingredients could take part in the regulation of nuclear receptor. Emodin has been proved to increase the mRNA level of PPAR*γ* and play a protective role in alcohol-mediated liver steatosis [37]. According to the activation of PPAR*γ* signaling pathway, emodin could also alleviate atherosclerosis followed by promoting cholesterol efflux [38] or play other roles though regulating inflammatory response [39, 40] and nitric oxide production [41]. Furthermore, emodin has also been reported to regulate the expression of LXR*α* in atherosclerosis [38] and melanogenesis [42]. Hyperin is one of the chief flavonoid components of Ericaceae, Guttifera, Leguminosae, and Celastraceae and could remarkably induce the expression of PPAR*γ* and attenuate inflammation of acute liver injury [43]. In addition, some studies also reported that rhein could target PPAR*γ* signaling pathway and play anti-inflammatory activity [44, 45]. Moreover, rhein has been confirmed to ameliorate NAFLD and obesity and recover metabolic disorders through directly binding to LXR*α* [46, 47]. Thus, those key ingredients in JZD might improve NAFLD via regulating the nuclear receptors.

In conclusion, the multicomponent and multitarget characteristics of the therapeutic effects of JZD against NAFLD were effectively elucidated through network pharmacology approach and experimental validation. Nuclear receptor transcription and lipid metabolism regulation were found as the core molecular mechanisms by which JZD alleviated NAFLD. Therefore, our study will provide the scientific evidences of the clinical efficacy of JZD against NAFLD.

## Figures and Tables

**Table 1:** The herbs of JZD.

Chinese name	Pharmaceutical name	Botanical plant name	English name
Gua Lou	Trichosanthis Fructus	*Trichosanthes kirilowii* Maxim.	Snakegourd fruit
Ze Xie	Alismatis Rhizoma	*Alisma orientale* (Sam.) Juzep	Oriental Waterplantain Rhizome
Dang Gui	Angelicae Sinensis Radix	*Angelica sinensis* (Oliv.) Diels	Chinese Angelica
Shan Zha	Crataegi Fructus	*Crataegus pinnatifida* Bge.	Hawthorn fruit
He Shou Wu	Polygoni Multiflori Radix	*Polygonum multiflorum* Thunb.	Fleece flower root

**Table 2:** The overlapped genes between the target genes of active ingredients and NAFLD-related genes.

No.	Uniprot ID	Gene ID	Gene symbol	Gene full name
1	P54710	486	FXYD2	FXYD domain containing ion transport regulator 2
2	P06396	2934	GSN	Gelsolin
3	P28472	2562	GABRB3	Gamma-aminobutyric acid type A receptor Beta3 subunit
4	P07996	7057	THBS1	Thrombospondin 1
5	P54289	781	CACNA2D1	Calcium voltage-gated channel auxiliary subunit Alpha2delta 1
6	P04818	7298	TYMS	Thymidylate synthetase
7	P17302	2697	GJA1	Gap junction protein alpha 1
8	P55011	6558	SLC12A2	Solute carrier family 12 member 2
9	P07585	1634	DCN	Decorin
10	P08729	3855	KRT7	Keratin 7
11	P14210	3082	HGF	Hepatocyte growth factor
12	P13569	1080	CFTR	CF Transmembrane conductance regulator
13	Q96SL4	2882	GPX7	Glutathione peroxidase 7
14	P13500	6347	CCL2	C-C motif chemokine ligand 2
15	O94925	2744	GLS	Glutaminase
16	P21802	2263	FGFR2	Fibroblast growth factor receptor 2
17	Q99584	6284	S100A13	S100 calcium binding protein A13
18	Q13133	10062	NR1H3	Nuclear receptor subfamily 1 group H member 3
19	P13716	210	ALAD	Aminolevulinate dehydratase
20	P82251	11136	SLC7A9	Solute carrier family 7 member 9
21	Q8N159	162417	NAGS	N-Acetylglutamate synthase
22	P21549	189	AGXT	Alanine-glyoxylate and serine-pyruvate aminotransferase
23	P34896	6470	SHMT1	Serine hydroxymethyltransferase 1
24	Q08828	107	ADCY1	Adenylate cyclase 1
25	O43708	2954	GSTZ1	Glutathione S-transferase zeta 1
26	P51570	2584	GALK1	Galactokinase 1
27	P15104	2752	GLUL	Glutamate-ammonia ligase
28	Q14749	27232	GNMT	Glycine N-methyltransferase
29	P36222	1116	CHI3L1	Chitinase 3 like 1
30	Q8TED1	493869	GPX8	Glutathione peroxidase 8 (putative)
31	P11137	4133	MAP2	Microtubule associated protein 2
32	P00750	5327	PLAT	Plasminogen activator, tissue type
33	Q9H2S1	3781	KCNN2	Potassium calcium-activated channel subfamily N member 2
34	P04181	4942	OAT	Ornithine aminotransferase
35	Q07869	5465	PPARA	Peroxisome proliferator activated receptor alpha
36	P01130	3949	LDLR	Low density lipoprotein receptor
37	P09488	2944	GSTM1	Glutathione S-transferase mu 1
38	P09211	2950	GSTP1	Glutathione S-transferase pi 1
39	Q03181	5467	PPARD	Peroxisome proliferator activated receptor delta
40	P30711	2952	GSTT1	Glutathione S-transferase theta 1
41	P08263	2938	GSTA1	Glutathione S-transferase alpha 1
42	P03956	4312	MMP1	Matrix metallopeptidase 1
43	Q9P2W7	27087	B3GAT1	Beta-1,3-glucuronyltransferase 1
44	O60674	3717	JAK2	Janus kinase 2
45	P08700	3562	IL3	Interleukin 3
46	P12821	1636	ACE	Angiotensin I converting enzyme
47	P00734	2147	F2	Coagulation factor II, thrombin
48	Q8WTV0	949	SCARB1	Scavenger receptor class B member 1
49	P06576	506	ATP5F1B	ATP synthase F1 subunit beta
50	O14594	1463	NCAN	Neurocan
51	P24298	2875	GPT	Glutamic-pyruvic transaminase
52	P01375	7124	TNF	Tumor necrosis factor
53	P37231	5468	PPARG	Peroxisome proliferator activated receptor gamma
54	O00206	7099	TLR4	Toll-like receptor 4
55	P06213	3643	INSR	Insulin receptor
56	P00505	2806	GOT2	Glutamic-oxaloacetic transaminase 2
57	P01584	3553	IL1B	Interleukin 1 beta
58	P05019	3479	IGF1	Insulin-like growth factor 1
59	P41235	3172	HNF4A	Hepatocyte nuclear factor 4 alpha

## Data Availability

The data of our research can be acquired from the Supplementary Materials uploaded with this article.

## Abbreviations

AlogP: Water partition coefficient
AO: *Alisma orientale* (Sam.) Juzep
AS: *Angelica sinensis* (Oliv.) Diels
BBB: Blood-brain barrier
Caco-2: Caco-2 permeability
CP: *Crataegus pinnatifida* Bge
DEGs: Differentially expressed genes
DL: Drug-likeness
FBS: Fetal bovine serum
GEO: Gene Expression Omnibus
GO: Gene Ontology
Hdon/Hacc: Number of hydrogen bond donors and receptors
HL: Half-life
HNF4*α*: Hepatocyte nuclear factor 4*α*
IGF: Insulin-like growth factor
IGFBPs: Insulin-like growth factor binding proteins
JZD: Jiangzhi Decoction
LXR*α*: Liver X receptor *α*
MW: Molecular weight
NAFLD: Nonalcoholic fatty liver disease
NASH: Nonalcoholic steatohepatitis
OA: Oleic acid
OB: Human oral bioavailability
PM: *Polygonum multiflorum* Thunb
PPARs: Peroxisome proliferator-activated receptors
PPI: Protein-protein interaction
qRT-PCR: Quantitative real-time PCR
SD: Standard deviation
TC: Cholesterol
TCM: Traditional Chinese medicine
TCMID: Traditional Chinese Medicine Integrated Database
TCMSP: Traditional Chinese Medicine Systems Pharmacology
TG: Triglyceride
TK: *Trichosanthes kirilowii* Maxim.

## Supplementary Materials

Supplementary 1 Table S1: 147 active ingredients found in Jiangzhi Decoction (JZD). The active ingredients were obtained from both Traditional Chinese Medicine Systems Pharmacology (TCMSP) database (http://lsp.nwu.edu.cn/tcmsp.php) according to the ADME principle: drug − likeness (DL) > 0.18 and Traditional Chinese Medicine Integrated Database (TCMID) (http://www.megabionet.org/tcmid/).

Supplementary 2 Table S2: 1285 targets of active ingredients of JZD. The targets were identified using ChemMapper database (http://lilab.ecust.edu.cn/chemmapper/) according to the criteria of 3D structure similarity above 1.0 and prediction score above 0.

Supplementary 3 Table S3: 401 nonalcoholic fatty liver disease- (NAFLD-) related genes. Some of NAFLD-related genes were searched from DisGeNET database (http://www.disgenet.org/web/DisGeNET/menu/home) with the top 30% of genes selected and the others from Microarray data GSE31803 and GSE49541 in Gene Expression Omnibus (GEO) database (http://www.ncbi.nlm.nih.gov/geo/) with a cutoff value of *P* < 0.05 and fold change ∣FC | ≥3.
